# (*E*,*E*)-2,5-Bis(5-chloro-2-methoxyphenyl)-3,4-diazahexa-2,4-diene

**DOI:** 10.1107/S1600536809048351

**Published:** 2009-11-25

**Authors:** Jian-Guo Chang, Jie Lu, Ren-Gao Zhao

**Affiliations:** a Department of Materials Science and Chemical Engineering, Taishan University, 271021 Taian, Shandong, People’s Republic of China; b Department of Architecture and Mechanical Engineering, Taishan University, 271021 Taian, Shandong, People’s Republic of China

## Abstract

The title compound, C_18_H_18_Cl_2_N_2_O_2_, was synthesized by the reaction of 1-(5-chloro-2-methoxy­phen­yl)ethanone with hydrazine hydrate. The mol­ecule lies on a crystallographic twofold axis passing through the mid-point of the N—N bond with one half-mol­ecule in the asymmetric unit. The dihedral angle between the two aromatic rings is 44.33 (4)°. In the crystal, inter­molecular C—H⋯O inter­actions link the mol­ecules into columns along the *c* axis

## Related literature

For azine compounds containing both a diimine linkage and N—N bonding, see: Kesslen *et al.* (1999 ▶); Kundu *et al.* (2005 ▶). For related structures, see: Glaser *et al.* (1995 ▶); Hunig *et al.* (2000 ▶).
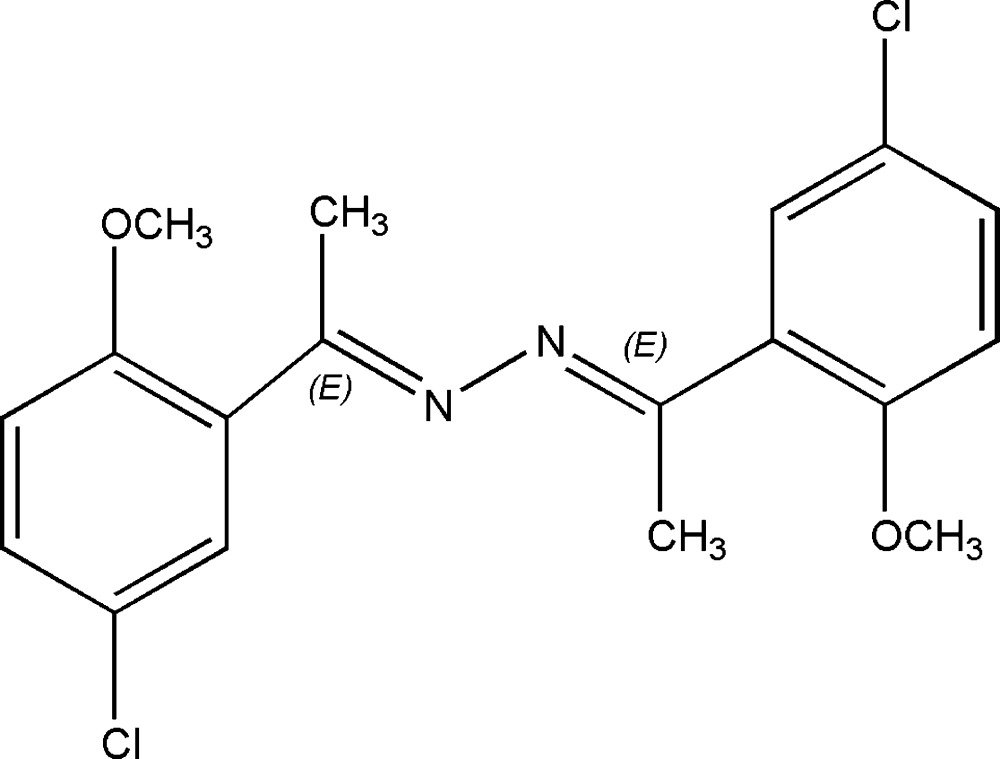


## Experimental

### 

#### Crystal data

C_18_H_18_Cl_2_N_2_O_2_
*M* *_r_* = 365.24
Orthorhombic, 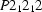

*a* = 7.9030 (19) Å
*b* = 27.862 (7) Å
*c* = 3.9819 (10) Å
*V* = 876.8 (4) Å^3^
*Z* = 2
Mo *K*α radiation
μ = 0.38 mm^−1^
*T* = 295 K
0.22 × 0.16 × 0.12 mm

#### Data collection

Bruker APEXII CCD area detector diffractometer
Absorption correction: multi-scan (*SADABS*; Sheldrick, 2003 ▶) *T*_min_ = 0.921, *T*_max_ = 0.956
4469 measured reflections
1566 independent reflections
1417 reflections with *I* > 2σ(*I*)
*R*_int_ = 0.019

#### Refinement

*R*[*F* ^2^ > 2σ(*F* ^2^)] = 0.036
*wR*(*F* ^2^) = 0.129
*S* = 1.01
1566 reflections
111 parameters
H-atom parameters constrained
Δρ_max_ = 0.12 e Å^−3^
Δρ_min_ = −0.22 e Å^−3^
Absolute structure: Flack (1983 ▶), 592 Friedel pairs
Flack parameter: 0.08 (12)

### 

Data collection: *APEX2* (Bruker, 2005 ▶); cell refinement: *SAINT* (Bruker, 2005 ▶); data reduction: *SAINT*; program(s) used to solve structure: *SHELXS97* (Sheldrick, 2008 ▶); program(s) used to refine structure: *SHELXL97* (Sheldrick, 2008 ▶); molecular graphics: *SHELXTL* (Sheldrick, 2008 ▶); software used to prepare material for publication: *SHELXTL*.

## Supplementary Material

Crystal structure: contains datablocks global, I. DOI: 10.1107/S1600536809048351/fl2273sup1.cif

Structure factors: contains datablocks I. DOI: 10.1107/S1600536809048351/fl2273Isup2.hkl

Additional supplementary materials:  crystallographic information; 3D view; checkCIF report

## Figures and Tables

**Table 1:** Hydrogen-bond geometry (Å, °)

*D*—H⋯*A*	*D*—H	H⋯*A*	*D*⋯*A*	*D*—H⋯*A*
C9—H9*B*⋯O1^i^	0.96	2.68	3.521 (3)	146

Symmetry code: (i) 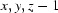.

**Fig. 1.**
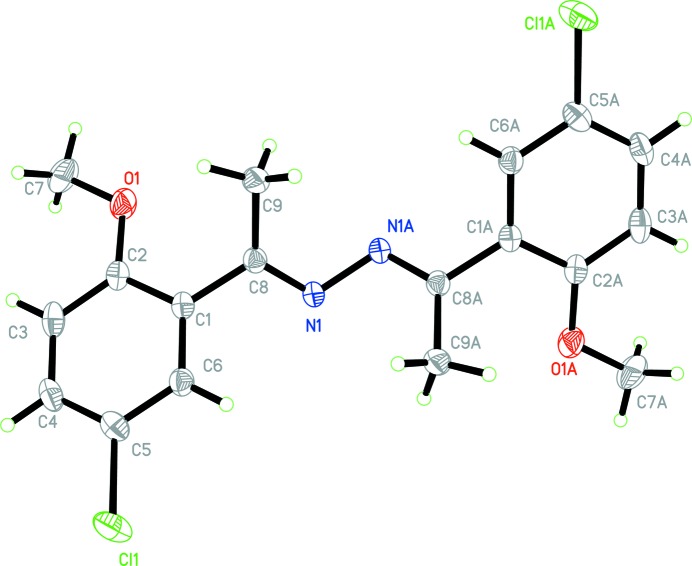
The molecular structure of compound (I), showing the atom-numbering scheme. Displacement ellipsoids are drawn at the 30% probability level.

**Fig. 2.**
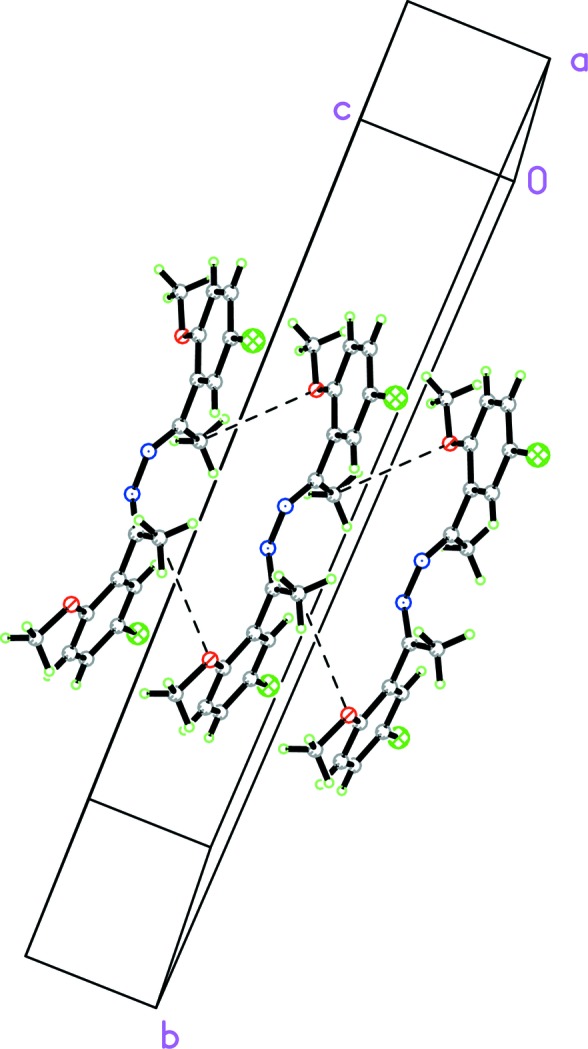
The crystal packing of (I) , Dashed lines show intermolecular C—H···O interactions .

## supplementary crystallographic information

### Comment

Recently, a number of azine compounds containing both a diimine linkage and N—N bonding have been investigated in terms of their crystallography and coordination chemistry (Kundu *et al.*, 2005; Kesslen *et al.*, 1999). As an extension of the work on the structural characterization of azine derivatives, the title compound, (I),was synthesized and its crystal structure is reported here.

The molecule lies on a crystallogrpahic 2-fold axis passing through the mid-point of the N-N bond to give 1/2 molecule per asymmetric unit. (Fig. 1). The dihedral angle between the two aromatic rings is 44.33 (4)°. The N atom and the phenyl ring liie on opposite side of the C8=N1 bond to give an (E, E) conformation with respect to the C8=N1 bond ( and its symmetry related C8*a*=N1a double bond (Fig. 1.). This configuration agrees with those commonly found in similar compounds (Glaser *et al.*, 1995; Hunig *et al.*, 2000). Intermolecular C—H···O interactions link the molecules into columns along the c axis (Table 1, Fig. 2).

### Experimental

An ethanol solution (30 ml) of hydrazine (0.02 mol) and 1-(5-chloro-2-methoxyphenyl)ethanone (0.04 mol) was refluxed and stirred for 6 h; the mixture was cooled and the resulting solid product, (I), was collected by filtration. Colourless crystals suitable for single-crystal X-ray diffraction were grown by slow evaporation of a solution in acetone.

### Refinement

All H atoms were positioned geometrically and treated as riding on their parent atoms,with *C*—*H*(methyl) = 0.96 Å, C—H(aromatic) = 0.93 Å, and with *U*_iso_(H) =1.5*U*_eq_(C_methyl_) and 1.2*U*_eq_(C_aromatic_).

### Crystal data

**Table:**

C_18_H_18_Cl_2_N_2_O_2_	*F*(000) = 380
*M**_r_* = 365.24	*D*_x_ = 1.383 Mg m^−^^3^
Orthorhombic, *P*2_1_2_1_2	Mo *K*α radiation, λ = 0.71073 Å
Hall symbol: P 2 2ab	Cell parameters from 2313 reflections
*a* = 7.9030 (19) Å	θ = 2.7–27.8°
*b* = 27.862 (7) Å	µ = 0.38 mm^−^^1^
*c* = 3.9819 (10) Å	*T* = 295 K
*V* = 876.8 (4) Å^3^	Block, colourless
*Z* = 2	0.22 × 0.16 × 0.12 mm

### Data collection

**Table:**

Bruker APEXII CCD area detector diffractometer	1566 independent reflections
Radiation source: fine-focus sealed tube	1417 reflections with *I* > 2σ(*I*)
graphite	*R*_int_ = 0.019
phi and ω scans	θ_max_ = 25.0°, θ_min_ = 1.5°
Absorption correction: multi-scan (*SADABS*; Sheldrick, 2003)	*h* = −9→8
*T*_min_ = 0.921, *T*_max_ = 0.956	*k* = −33→32
4469 measured reflections	*l* = −4→4

### Refinement

**Table:**

Refinement on *F*^2^	Secondary atom site location: difference Fourier map
Least-squares matrix: full	Hydrogen site location: inferred from neighbouring sites
*R*[*F*^2^ > 2σ(*F*^2^)] = 0.036	H-atom parameters constrained
*wR*(*F*^2^) = 0.129	*w* = 1/[σ^2^(*F*_o_^2^) + (0.1019*P*)^2^ + 0.021*P*] where *P* = (*F*_o_^2^ + 2*F*_c_^2^)/3
*S* = 1.01	(Δ/σ)_max_ < 0.001
1566 reflections	Δρ_max_ = 0.12 e Å^−^^3^
111 parameters	Δρ_min_ = −0.22 e Å^−^^3^
0 restraints	Absolute structure: Flack (1983), 592 Friedel pairs
Primary atom site location: structure-invariant direct methods	Flack parameter: 0.08 (12)

### Special details

**Table:**

Geometry. All esds (except the esd in the dihedral angle between two l.s. planes) are estimated using the full covariance matrix. The cell esds are taken into account individually in the estimation of esds in distances, angles and torsion angles; correlations between esds in cell parameters are only used when they are defined by crystal symmetry. An approximate (isotropic) treatment of cell esds is used for estimating esds involving l.s. planes.
Refinement. Refinement of F^2^ against ALL reflections. The weighted R-factor wR and goodness of fit S are based on F^2^, conventional R-factors R are based on F, with F set to zero for negative F^2^. The threshold expression of F^2^ > 2sigma(F^2^) is used only for calculating R-factors(gt) etc. and is not relevant to the choice of reflections for refinement. R-factors based on F^2^ are statistically about twice as large as those based on F, and R- factors based on ALL data will be even larger.

### Fractional atomic coordinates and isotropic or equivalent isotropic displacement parameters (Å^2^)

**Table:**

	*x*	*y*	*z*	*U*_iso_*/*U*_eq_	
Cl1	0.35358 (10)	0.29469 (2)	0.1116 (2)	0.0643 (3)	
O1	0.9306 (2)	0.40920 (6)	0.5876 (6)	0.0520 (5)	
N1	0.5044 (3)	0.47464 (6)	0.4965 (5)	0.0367 (5)	
C1	0.6541 (3)	0.40440 (7)	0.3671 (6)	0.0333 (5)	
C2	0.8000 (3)	0.38071 (8)	0.4798 (6)	0.0373 (6)	
C3	0.8050 (4)	0.33068 (10)	0.4871 (7)	0.0487 (7)	
H3	0.9006	0.3150	0.5674	0.058*	
C4	0.6670 (3)	0.30434 (8)	0.3743 (7)	0.0468 (7)	
H4	0.6702	0.2710	0.3770	0.056*	
C5	0.5261 (4)	0.32784 (9)	0.2588 (7)	0.0429 (6)	
C6	0.5175 (3)	0.37744 (8)	0.2532 (6)	0.0381 (6)	
H6	0.4208	0.3927	0.1736	0.046*	
C7	1.0770 (4)	0.38654 (12)	0.7221 (9)	0.0611 (8)	
H7A	1.0453	0.3671	0.9111	0.092*	
H7B	1.1565	0.4106	0.7929	0.092*	
H7C	1.1279	0.3667	0.5532	0.092*	
C8	0.6407 (3)	0.45789 (8)	0.3653 (6)	0.0330 (5)	
C9	0.7746 (3)	0.48801 (9)	0.2056 (7)	0.0413 (6)	
H9A	0.7244	0.5081	0.0369	0.062*	
H9B	0.8580	0.4676	0.1039	0.062*	
H9C	0.8274	0.5077	0.3734	0.062*	

### Atomic displacement parameters (Å^2^)

**Table:**

	*U*^11^	*U*^22^	*U*^33^	*U*^12^	*U*^13^	*U*^23^
Cl1	0.0758 (5)	0.0434 (4)	0.0738 (5)	−0.0196 (3)	−0.0095 (5)	−0.0004 (4)
O1	0.0404 (9)	0.0499 (10)	0.0656 (13)	0.0104 (8)	−0.0170 (10)	−0.0051 (10)
N1	0.0331 (10)	0.0273 (9)	0.0497 (12)	0.0040 (8)	−0.0013 (9)	0.0009 (9)
C1	0.0368 (12)	0.0316 (12)	0.0315 (11)	0.0055 (9)	0.0014 (11)	0.0012 (9)
C2	0.0404 (13)	0.0386 (12)	0.0329 (12)	0.0102 (10)	−0.0014 (10)	−0.0024 (10)
C3	0.0568 (16)	0.0408 (13)	0.0485 (15)	0.0213 (12)	−0.0006 (13)	0.0047 (12)
C4	0.0618 (17)	0.0286 (11)	0.0500 (15)	0.0080 (11)	0.0084 (15)	0.0008 (11)
C5	0.0567 (16)	0.0327 (12)	0.0392 (13)	−0.0037 (12)	0.0022 (12)	0.0010 (10)
C6	0.0382 (13)	0.0343 (12)	0.0420 (13)	0.0049 (10)	−0.0002 (11)	0.0035 (10)
C7	0.0409 (14)	0.082 (2)	0.0605 (19)	0.0158 (15)	−0.0150 (13)	−0.0022 (17)
C8	0.0340 (11)	0.0311 (11)	0.0337 (11)	0.0024 (9)	−0.0040 (11)	−0.0019 (9)
C9	0.0400 (12)	0.0362 (13)	0.0475 (15)	−0.0018 (11)	0.0057 (11)	−0.0039 (11)

### Geometric parameters (Å, °)

**Table:**

Cl1—C5	1.748 (3)	C4—C5	1.371 (4)
O1—C2	1.371 (3)	C4—H4	0.9300
O1—C7	1.422 (3)	C5—C6	1.384 (3)
N1—C8	1.285 (3)	C6—H6	0.9300
N1—N1^i^	1.415 (3)	C7—H7A	0.9600
C1—C6	1.391 (3)	C7—H7B	0.9600
C1—C2	1.403 (3)	C7—H7C	0.9600
C1—C8	1.494 (3)	C8—C9	1.493 (3)
C2—C3	1.395 (4)	C9—H9A	0.9600
C3—C4	1.389 (4)	C9—H9B	0.9600
C3—H3	0.9300	C9—H9C	0.9600
			
C2—O1—C7	118.2 (2)	C5—C6—H6	120.1
C8—N1—N1^i^	113.8 (2)	C1—C6—H6	120.1
C6—C1—C2	119.2 (2)	O1—C7—H7A	109.5
C6—C1—C8	118.9 (2)	O1—C7—H7B	109.5
C2—C1—C8	121.9 (2)	H7A—C7—H7B	109.5
O1—C2—C3	123.4 (2)	O1—C7—H7C	109.5
O1—C2—C1	116.53 (19)	H7A—C7—H7C	109.5
C3—C2—C1	120.0 (2)	H7B—C7—H7C	109.5
C4—C3—C2	119.9 (2)	N1—C8—C9	124.3 (2)
C4—C3—H3	120.0	N1—C8—C1	114.8 (2)
C2—C3—H3	120.0	C9—C8—C1	120.9 (2)
C5—C4—C3	119.6 (2)	C8—C9—H9A	109.5
C5—C4—H4	120.2	C8—C9—H9B	109.5
C3—C4—H4	120.2	H9A—C9—H9B	109.5
C4—C5—C6	121.5 (3)	C8—C9—H9C	109.5
C4—C5—Cl1	119.57 (19)	H9A—C9—H9C	109.5
C6—C5—Cl1	118.9 (2)	H9B—C9—H9C	109.5
C5—C6—C1	119.7 (2)		
			
C7—O1—C2—C3	−1.7 (4)	C4—C5—C6—C1	−0.1 (4)
C7—O1—C2—C1	176.4 (2)	Cl1—C5—C6—C1	−179.54 (19)
C6—C1—C2—O1	179.7 (2)	C2—C1—C6—C5	1.3 (4)
C8—C1—C2—O1	−0.2 (3)	C8—C1—C6—C5	−178.8 (2)
C6—C1—C2—C3	−2.1 (4)	N1^i^—N1—C8—C9	−3.8 (3)
C8—C1—C2—C3	178.0 (2)	N1^i^—N1—C8—C1	179.13 (16)
O1—C2—C3—C4	179.8 (2)	C6—C1—C8—N1	48.8 (3)
C1—C2—C3—C4	1.7 (4)	C2—C1—C8—N1	−131.3 (2)
C2—C3—C4—C5	−0.5 (4)	C6—C1—C8—C9	−128.4 (2)
C3—C4—C5—C6	−0.3 (4)	C2—C1—C8—C9	51.5 (3)
C3—C4—C5—Cl1	179.2 (2)		

Symmetry codes: (i) −*x*+1, −*y*+1, *z*.

### Hydrogen-bond geometry (Å, °)

**Table:**

*D*—H···*A*	*D*—H	H···*A*	*D*···*A*	*D*—H···*A*
C9—H9B···O1^ii^	0.96	2.68	3.521 (3)	146

Symmetry codes: (ii) *x*, *y*, *z*−1.
